# Linking Formal Child Care Characteristics to Children’s Socioemotional Well-Being: A Comparative Perspective

**DOI:** 10.1007/s10826-018-1185-2

**Published:** 2018-07-20

**Authors:** Melissa Verhoef, Anke C. Plagnol, Vanessa May

**Affiliations:** 1 Department of Sociology, Utrecht University, P.O. Box 80140, Utrecht, TC 3508 The Netherlands; 2 Department of Psychology, City, University of London, London, UK; 3 Morgan Centre for Research into Everyday Lives, Department of Sociology, University of Manchester, Manchester, UK

**Keywords:** Child-care arrangements, Child well-being, Parental employment, Comparative research

## Abstract

Most research on formal child care and children’s outcomes has focused on single countries. We, however, contend that policy context may moderate the association between formal child care characteristics and children’s socioemotional well-being. We examined this by comparing the Netherlands, Finland and the UK; three countries that differ regarding family policies. Of these three countries, Finland was recently ranked highest (ranked 1st) with regards to quality of child care in a recent analysis by the Economist, followed by the UK (ranked 3rd) and then the Netherlands (ranked 7th). We hypothesized that children who attend child-care settings in countries with higher-quality formal child-care provision would generally show better socioemotional outcomes. Data from the comparative ‘Families 24/7’ survey were used, including 990 parents with children aged 0–12. We distinguished between two age groups in our analysis. Results indicated that, compared to the UK, longer hours in formal care were less beneficial in the Netherlands. Furthermore, spending time in formal care during nonstandard hours was more harmful for children in Finland compared to the UK. Lastly, receiving care from multiple caregivers was more disruptive for British children than for Dutch children. No differences were found between Finland and the Netherlands.

## Introduction

Family life has undergone significant changes in the past few decades. The formerly prominent male breadwinner model has weakened in many Western societies, as a large proportion of mothers have entered the labor market (Crompton et al. 2007). Given that mothers no longer stay at home by default to care for their children, the demand for formal child care—i.e., care provided by professionals (Zinsser 2001)—has increased, making the provision of formal care an integral part of contemporary welfare states (Mahon 2002).

Even though the use of formal child care is relatively common in Western countries, considerable country variation exists (OECD Family Database 2015). Family policies are important in this case. For example, increases in child-care subsidies have been found to affect formal care enrolment positively (Greenberg 2010). High levels of child-care subsidies have even been linked to lower child poverty and child mortality (Engster and Stensöta 2011). Moreover, greater governmental investments in formal care and more stringent regulations regarding educational requirements for staff have been shown to increase the quality of formal care (Rigby et al. 2007). High-quality child care has in turn been associated with better child outcomes (Broekhuizen et al. 2016), indicating that family policies matter not only for child-care enrolment, but also for child well-being. Whereas the existing literature does provide insight into country differences in the use of formal child care (Kröger 2010; Mamolo et al. 2011), studies on formal child care and child outcomes tend to focus on single countries.

The link between the characteristics of formal child care and children’s socioemotional well-being can be explained with the help of Bronfenbrenner’s (1979) ecological systems theory. This theory supposes that individuals develop in an environment that consists of multiple, overlapping systems. Systems closest to the individual are called microsystems, in which individuals can readily engage in face-to-face interactions. Examples of such systems are the family, child-care setting or peer group. Given that many children spend time in formal child care during childhood, the formal child-care setting in which they are enrolled makes up an important microsystem in their lives. As explained by Bronfenbrenner (1979), activities and interconnectedness within the microsystem constitute building blocks for the way in which the microsystem affects the individual. When problems occur with these building blocks, individuals are likely to be negatively affected. Such reasoning explains why formal care characteristics are associated with children’s socioemotional well-being. For instance, when children spend time in formal care during nonstandard hours, there are likely fewer children present, which makes it difficult for these children to interact with peers. This poses problems for interconnectedness. Children may even feel isolated, because they have to sleep in an environment that is not their house while their peers go home in the evening. Even young children may feel that this is outside the norm. Consequently, the lower level of interconnectedness may result in lower socioemotional well-being.

Microsystems are embedded in several other systems, of which the macrosystem is the broadest, overarching system (Bronfenbrenner 1979). The macrosystem includes the values, policies and customs of an extended social structure. Through social policy, resources can be provided that enable the processes in the lower-level systems to work as effectively as possible (Bronfenbrenner and Ceci 1994). Regarding child care, this applies to the specific family policy context in which child-care settings are embedded. Policies that are aimed at providing high-quality care are, therefore, expected to improve the processes occurring in the child-care settings. Studies that test interactions between micro- and macrosystems related to formal child care are, however, scarce as most studies on associations between formal child care and child outcomes focus on a single country and do therefore not allow an analysis of how the policy context (i.e., macrosystem) may moderate the association between formal child care (i.e., microsystem) and children’s well-being. However, it is possible that the association between formal child-care characteristics and children’s socioemotional well-being depends on the country-specific context in which formal care is provided. Given the better child outcomes in high-quality care, children likely thrive more in countries in which family policies provide better support for parents and ensure high-quality child care.

Prior research has demonstrated that enrolment in formal care is positively associated with children’s cognitive development, reflected in improved cognitive and language skills over time (e.g., Votruba-Drzal et al. 2013; Weiland and Yoshikawa 2013). Moreover, the interactions that children have with peers and non-kin adults are thought to increase children’s sociability (Howes 2011). Socioemotional child outcomes are, however, generally less positive, as reflected in higher levels of behavioral problems (e.g., Magnuson et al. 2007; McCartney et al. 2010). This is especially troublesome because early-life experiences in child care have been shown to have long-lasting effects, for example, on school outcomes and health (Campbell et al. 2014; Nores and Barnett 2010). It is therefore important to further investigate to what extent different aspects of child-care use may safeguard children from negative socioemotional outcomes; for example the number of hours spent in child care, attendance during nonstandard hours, and variability in care arrangements.

Starting with the amount of time children spend in formal care, research has demonstrated that more hours in care are related to higher levels of externalizing behavior (Loeb et al. 2007), enduring even until age 15 (Vandell et al. 2010), whereas there seems to be no association with internalizing behavior. Regarding prosocial behavior, enrolment in formal care has been found to positively affect the sociability of children during both their time in formal care and later development (Abner et al. 2013; Howes 2011), implying that more time in formal care may positively affect children’s social development.

Because the share of parents who work during evenings, nights and weekends (i.e., nonstandard hours) has expanded in Western societies (Bünning and Pollmann-Schult 2016; Presser 2003), it has become increasingly important to consider the scheduling of the hours that children spend in formal care. Yet, studies on this topic are scarce. The limited available research reveals that children who are in formal care overnight show delays in motor, intelligence and social development, compared to children who are in formal care only during the day or evening (Anme and Segal 2003). Furthermore, formal care outside standard hours has been associated with decreased social competency and increased behavioral and emotional problems, compared to formal care during the 6 am to 6 pm timeframe (Boyd-Swan 2015).

The number of care arrangements—a measure of variability within formal child care—has been found to be positively associated with more externalizing and internalizing problems and less prosocial behavior, with results being most severe for girls and children under the age of three (Morrissey 2009). In addition, a study by Claessens and Chen (2013) shows higher levels of conduct problems and lower prosocial behavior among children under the age of five enrolled in multiple care arrangements.

Some studies have identified that formal care seems to affect younger children more strongly than older children. For instance, Morrissey (2009) found more behavioral problems and less prosocial behavior among children aged below three. Phillips and Adams (2001), however, argue that younger children may not only be at increased risk for adverse outcomes, but also experience enhanced learning. A recent literature review on the effects of formal care on child development also points in this direction, given the mixed findings among the group of children aged zero to three (Melhuish et al. 2015). Although it remains debatable in which direction younger children are affected more strongly by formal child care than older children, existing studies are clear in demonstrating that the association between formal care and child well-being is not similar across age groups.

Most of the studies cited above, however, all focus on a single country and do therefore not provide insight into what role country-specific conditions, such as family policies (the macrosystem in Bronfenbrenner’s ecological systems theory), play in the association between formal child care (the microsystem) and child outcomes. A cross-national perspective may provide insight into the relevance of the country-specific context in which formal child care is provided; especially if the countries that are compared employ different family policies leading to considerable differences in child-care characteristics.

The Netherlands, Finland and the UK represent distinct examples on a spectrum of child-care provisions found in Europe with regards to child-care availability, up-take and quality. The summary of child-care characteristics and use in Table 1 shows that the up-take of formal child care, especially for very young children, differs considerably between these countries, likely due to varying levels of financial support with child-care costs provided by the government and thus differing impact on out-of-pocket costs for parents. Differences in the length of paid parental leave may also allow parents in Finland to stay home with their children for a longer time than in the Netherlands and in the UK and thus provide them with more choice with regards to electing formal child care for their young children or parental care. All three countries perform well in the quality ranking of the 2012 Starting Well Index of the Economist Intelligence Unit; each country is listed in the top 10 out of 45 countries that are included in the index (Watson 2012). However, child-care staff are generally better educated in Finland than in the other two countries included here. One would therefore expect that children’s socioemotional outcomes may generally be better in Finland, but child-care characteristics in these three countries also differ in other aspects, often depending on what policymakers consider to be the primary purpose of child care.

**Table 1:** Child care characteristics and use in the Netherlands, Finland, and the UK

	Netherlands	Finland	UK
Help with costs of formal child care	Provided to employed parents	Municipal child care heavily subsidized by government, depending on income	Modest help in the form of tax credits and child-care vouchers. 15 h of free child care after child’s third birthday
Out-of-pocket net cost of formal childcare for a two-earner couple family as a % of family net income^a^	21.3%	17.9%	40.8%
Enrolment in formal care: children aged 2 and under^a^	56%	28%	34%
Enrolment in formal care: children 3–5 years old^a^	92%	74%	94%
Rank in Starting Well Index (Quality), 2012^b^	7th	1st	3rd
Child-staff ratio	Range from 4:1 to 10:1	Range from 4:1 to 7:1	Range from 3:1 to 8:1; 13:1 allowed if qualified teacher is employed
Education requirements of child care staff	At least upper secondary-level education	At least upper secondary-level education, one in three staff must have a university degree in education or social services	At least half of staff have to hold at least lower secondary-level education. Child care center has to be managed by someone with a relevant upper secondary education

*Sources*: ^a^OECD Family Database (2015)

^b^Watson (2012; Table 4)

In the Netherlands, the state was not supposed to intervene with family life, but this changed when formal child care came to be viewed as a way to increase female employment, which led to large increases in the budget for child care (Van Hooren and Becker 2012). Dutch parents can choose from four types of formal child care, which are partly dependent on the age of the child. For preschoolers, parents can either choose center-based daycare, a playgroup, or a childminder, whereas the choice for school-aged children is between center-based out-of-school care or care by a childminder (Dutch Government 2014). Although most services used to operate during standard working hours (8 am to 6 pm) on weekdays only (EACEA 2009), in the past few years the child-care sector has recognized parents’ demands for child care outside office hours, making extended and 24-7 center-based child care available (De Jong 2013). Childminders also offer evening and weekend care to some extent, whereas the supply of night care is limited (Boogaard and Bollen 2014). Formal child-care services are targeted at working parents, as child-care benefits are provided only to them (Social and Economic Council of the Netherlands 2016). Dutch formal child care is therefore first and foremost a labor market instrument. The costs of Dutch formal child care are divided between working parents, the government (i.e., child-care benefits) and employers, where the governmental share is dependent on working parents’ income, ranging from 23.8 to 93 per cent of the costs (Social and Economic Council of the Netherlands 2016). On average, Dutch parents spend more than one-fifth of their net income on child care (OECD Family Database 2015).

Almost 56 per cent of Dutch children under the age of three are enrolled in formal care, as well as 92 per cent of Dutch children between three and five years (OECD Family Database 2014). Regulations exist to safeguard the quality of care that children receive, focusing on pedagogical aspects, such as developing social competence and offering emotional security. Despite existing regulations, there have been concerns about the quality of formal child care in the Netherlands (Fukkink et al. 2013). However, the Netherlands seems to be doing fairly well from an international perspective, as the country is ranked seventh on the quality dimension of the 2012 Starting Well Index of the Economist Intelligence Unit. This index compared 45 countries on several quality indicators, such as the child-staff ratio, curriculum guidelines and education requirements for staff (Watson 2012). For instance, child-staff ratios range from 4:1 to 10:1 in the Netherlands (De Hond et al. 2012) and formal child-care staff should hold at least an upper secondary education degree (Dutch Child Care Act 2016).

In contrast, the provision of child care in Finland is based on the principle of shared responsibility for the care and welfare of all children (Lamb and Ahnert 2006), so this is targeted not only at children of working parents. The Day Care Act of 1973 granted children over the age of three the universal right to formal child care, a right that in 1996 was extended to all children under school age (Alila 2015). The aims of this legislation were manifold, including the reconciliation of work and family and the provision of early education, but formal child care was also seen as a family and social policy tool. Parents can choose between private and municipal child care; the latter option is by far the most popular, with over 90 per cent of children in municipal child care, which is available in the form of child-care centers and family daycare (Alila 2015). At age six, most children go on to attend a year of preschool. The opening hours of child-care centers vary from ‘normal’ hours (7 am to 5 pm) to extended hours and 24-7 child care in day-and-night care centers. Formal care provision for school-aged children consists of before- and after-school activities organized by the municipality between 7 am to 5 pm (Finlex 2016). Child-care fees are heavily subsidized by the government, with the exact fee that parents pay dependent on their income and the number of children attending municipal child care (Finnish National Agency for Education 2016). Finnish parents spend about 18 per cent of their net income on child care (OECD Family Database 2015).

With 28 per cent of Finnish children aged under three being enrolled in formal child care, enrolment rates are relatively low. This is due to the popularity of the home care leave (Morgan and Zippel 2003). Enrolment rates are significantly higher for children aged over three, at 74 per cent (OECD Family Database 2014). Approximately 7 per cent of children enrolled in formal child care attend a day-and-night care center (Säkkinen 2014). The quality of the care that Finnish children receive has been evaluated as being high, especially concerning emotional and instructional support provided by caregivers (Pakarinen et al. 2010). This high quality of care is reflected in the Starting Well Index, as Finland is ranked first on the quality dimension (Watson 2012). Child-staff ratios range from 4:1 to 7:1 and child-care center staff must have at least an upper secondary-level qualification, and one in three of the staff at a child-care center must have a university degree in education or social services (Alila 2015).

Child care in the UK has traditionally been viewed as a private or individual concern. However, the country has moved from this point of view, now embracing the state’s role in ensuring access to high quality care, accompanied by major investments (British Department for Education 2013; Wincott 2006). Still, formal child care is mostly offered by private providers in the form of day nurseries, childminders, nannies and au pairs (NCT 2016). These child-care providers offer care for children ranging from young babies up to age 5. Children’s centers, which are under the control of local authorities, are also available, but usually not for children under two and often only part-time. In addition, all children are eligible for 15 h of free child care after their third birthday. Child-care services in the UK are usually only open during standard working hours (8 am to 5:30 pm). Nurseries that provide extended or even 24 h care do exist, although this is limited to specific locations, such as near hospitals or airports (Formby et al. 2004). Most parents in the UK receive only modest financial help to pay for formal care, in the form of tax credits or child-care vouchers. Some children from low-income households receive a number of free formal child-care hours after their second birthday. The costs for child care in the UK are higher than in other European countries, taking up over 40 per cent of parents’ net income (OECD Family Database 2015).

Enrolment statistics illustrate that 34 per cent of British children aged two and under are enrolled in formal child care. For children aged three to five years, enrolment rates are much higher: 94 per cent of this age group are enrolled in formal child care (OECD Family Database 2014). The quality of the care that British children receive is relatively high, as the UK is ranked third on the Starting Well Index (Watson 2012), indicating that the UK takes the middle position between the Netherlands and Finland. However, there have been worries about the quality of the British formal care system, especially in poorer areas (Lloyd and Penn 2010). Child-staff ratios range from 3:1 to 8:1, although 13:1 is allowed if the children are cared for by a qualified teacher (British Department for Education 2013). Educational requirements for staff prescribe that at least half of the staff at a child-care setting have to hold a lower secondary qualification and the provision has to be managed by someone with a relevant upper secondary qualification (Nutbrown 2012).

The above description of formal child-care systems in the Netherlands, Finland and the UK illustrates that there are marked differences between these countries. Based on Bronfenbrenner’s (1979) ecological systems theory, we contend that such differences may affect the extent to which formal care characteristics are associated with children’s socioemotional well-being. The current study takes a comparative perspective by analyzing children’s outcomes in these three countries, with a focus on three characteristics of child care that have been shown to affect socioemotional child well-being: the amount of time children spend in formal care, the scheduling of the hours spent in child care, and the number of care arrangements. In this study, socioemotional well-being is defined as a general emotional state, hereby tapping into the child’s behavioral and emotional strengths, as well as how the child responds to adversity. By paying attention to the social interactions of children, we take into account that this concept does not rely only on the individual characteristics of the child, but also on his/her relationships with their surroundings (e.g., Hamilton and Redmond 2010). We cover several dimensions of well-being, including behavioral problems and prosocial behavior. We argue that country-specific regulations for child-care settings matter for children’s socioemotional well-being and that children thrive more in countries with regulations that ensure high-quality child care. Given that the Finnish child-care system is ranked highest in terms of the quality of care, followed by the UK and then the Netherlands, we hypothesize that *spending more hours in formal child care will be most positive for the socioemotional well-being of Finnish children, slightly less positive for British children, and least positive for Dutch children* (H1). Furthermore, in Finland governmental regulations specifically address care during nonstandard hours, which is not the case in the Netherlands and the UK. However, in the Netherlands the supply of these services seems to be more common than in the UK, which may lead to better quality care during nonstandard hours due to increased options for parents and potential sharing of best practice between child-care settings. We therefore expect that *spending time in formal care during nonstandard hours will be least disruptive for children’s socioemotional well-being in Finland, somewhat more disruptive in the Netherlands, and most disruptive in the UK* (H2). In addition, we expect that variability in formal care providers, as measured by the number of care arrangements, is less detrimental to children’s outcomes in countries with more favorable child-care conditions. With respect to the three countries in our study, we therefore hypothesize that *increases in the number of caregivers will be least harmful for children’s* socioemotional well-being in Finland, followed by the UK, and most harmful in the Netherlands (H3). We focus on these three child-care characteristics in our analysis because the questionnaires that were employed in the data collection were completed by parents. We therefore had to rely on their knowledge of their children’s child-care arrangements, which restricted the set of possible measures of child-care characteristics. The three measures of child-care characteristics chosen here—i.e. hours spent in child care, spending time in child care during nonstandard hours, and number of caregivers—all relate to the extent of children’s relationships with nonparental caregivers. These social interactions thus all potentially challenge children’s behavioral and emotional strength, which may be reflected in their socioemotional well-being. In addition, we differentiate between two age groups: children between zero and two years old and children aged three and older.

## Method

### Participants

This study utilizes data from the Families 24/7 survey, a comparative survey of Dutch, Finnish and British working parents with children aged 0 to 12, which includes extensive information on the child-care arrangements of working parents, as well as multiple indicators for child well-being (see also Moilanen et al. 2016; Verhoef et al. 2016). A total of 1,294 parents completed the questionnaire. For this study, the total sample size was restricted in three ways. First, we excluded families without income from employment (*n* = 11). Second, we excluded 281 respondents who reported not having used formal child care in the week prior to the survey. Third, we excluded respondents who indicated living less than half of the time with the target child (*n* *=* 12). Our final sample consists of 990 respondents, including 318 Dutch, 359 Finnish and 313 British respondents.

Sample characteristics are presented in Table 2, separately per country. In each country, the majority of the sample comprised women. British respondents were on average 38.13 years old, slightly older than Finnish (35.14) and Dutch (35.67) respondents. Respondents had on average two children, with Finnish respondents (1.87) reporting a higher number of children than British respondents (1.70). In terms of educational background, almost three-quarters (73.58%) of the Dutch sample had completed tertiary education, which was also the case for 43.02 per cent of the Finnish sample and 82.32 per cent of the British sample. For Finland, this is in line with average parental education in the country, whereas the Dutch and British parents in our sample are more highly educated than the general population with children (OECD 2016). Respondents answered child-related questions about a specific child, for which there were no gender differences between the countries. The average age of the child was lower in the Netherlands (3.16) compared to Finland (4.13) and the UK (4.22). For the majority of the sample, the child lived with both biological parents, although this was slightly less likely in Finland than in the other two countries. Respondents from the UK reported most problems with arranging child care, followed by Finnish and Dutch respondents, respectively. The family’s financial situation was rated more favorably in the Netherlands as compared to Finland and the UK.

**Table 2:** Sample characteristics separately per country (*N* = 990)

	NL (*n* = 318)		FI (*n* = 359)		UK (*n* = 313)		Mean difference test
	*M* (SD)	Range	*M* (SD)	Range	*M* (SD)	Range	
Gender respondent (1 = *female*)	0.86	0–1	0.82	0–1	0.85	0–1	*ns*
Age respondent (in years)	35.67 (5.34)	22–54	35.14 (5.33)	23–51	38.13 (0.76)	25–54	UK > FI & NL^***^
Number of children	1.80 (0.84)	1–8	1.87 (0.83)	1–5	1.70 (0.67)	1–4	FI > UK^*^
Education respondent (1 = *tertiary*)	0.74	0–1	0.43	0–1	0.82	0–1	UK > NL > FI^***^
Gender child (1 = *girl*)	0.55	0–1	0.53	0–1	0.47	0–1	*ns*
Age child (in years)	3.16 (2.32)	0–12	4.13 (1.88)	1–12	4.22 (2.92)	0–12	FI & UK > NL^***^
Child lives with both biological parents (1 = *yes*)	0.92	0–1	0.79	0–1	0.89	0–1	NL & UK > FI^***^
Problems with arranging child care (1 = *yes*)	0.07	0–1	0.30	0–1	0.47	0–1	UK > FI > NL^***^
Family’s financial situation	6.36 (1.91)	0–10	5.45 (2.15)	0–10	5.38 (2.15)	0–10	NL > FI & UK^***^

SD is not reported for dichotomous variables

*p *<* 0.05. ^***^p < 0.001

### Procedure

In all three countries, child-care organizations, unions and employers were approached, via letter or email, with the request to promote the study. Cooperating institutions were provided with posters, newsletters and leaflets about the study, which they could distribute among their costumers, members or employees. Only the Dutch child-care settings were selected randomly; all other institutions were recruited via convenience sampling. As day-and-night child-care organizations are more common in Finland than in the Netherlands and the UK, Finnish parents who use formal care during nonstandard hours are likely to be overrepresented.

The survey contained questions about the respondent, the respondent’s partner and a so-called target child, which refers to the child closest to age four. For example, if a respondent had a child aged seven and a child aged three, the respondent was asked to reply to a specific set of questions with the three-year-old in mind. All questions were first prepared in English; translation into Dutch or Finnish occurred via the use of existing national surveys or via back-translation. Data were collected via a web survey between November 2012 and January 2013. Because of our sampling method, we were not able to determine the response rate. Our method of data collection further required internet access to participate in the study, which may exclude potential respondents from lower socio-economic backgrounds.

### Measures

#### Children’s socioemotional well-being

We examined children’s socioemotional well-being in two age groups. First, for children aged three or older, we used the parent report version of the Strengths and Difficulties Questionnaire (SDQ) (Goodman 1997). This questionnaire contains 25 items, ranging from 1 (*not true*) to 3 (*certainly true*). Although the SDQ encompasses five different subscales, research has indicated that these subscales are less reliable in low-risk and general samples (Goodman et al. 2010). We therefore use the broader internalizing (e.g., “My child gets picked on or bullied by other children”) and externalizing (e.g., “My child often gets really angry and has temper tantrums”) subscales to tap into children’s difficulties, with higher scores being indicative of lower socioemotional well-being. Cronbach’s alpha of the internalizing subscale was 0.673 in the Netherlands, 0.638 in Finland and 0.681 in the UK, and respectively 0.792, 0.804 and 0.789 for the externalizing subscale. In addition, we use the prosocial subscale (e.g., “My child is helpful if someone is hurt, upset or feeling ill”) to cover children’s positive development; higher scores are indicative of higher socioemotional well-being. Cronbach’s alpha equals 0.652 in the Netherlands, 0.690 in Finland and 0.687 in the UK. Some of the multi-item measures included in the analysis are therefore considered to present questionable rather than acceptable internal reliability as their Cronbach’s alpha values fall in the 0.6–0.7 range.

Second, for children aged zero to two the EAS Temperament Survey (Buss and Plomin 1984) was used. Temperament, although considered as an individual’s innate personality, becomes visible in early childhood, for instance by the way children react to their environment (e.g., Griggs et al. 2009). The EAS measures temperament by assessing these visible behavioral characteristics of children. Parents were presented with 15 items, with answer categories varying from 1 (*not characteristic/typical of my child*) to 5 (*very characteristic/typical of my child*). The items encompass three subscales, namely emotionality (e.g., “My child reacts intensely when upset”), activity (e.g., “My child is always on the go”) and shyness (e.g., “My child takes a long time to warm up to strangers”). For all subscales, higher scores are indicative of lower well-being. Cronbach’s alpha of the emotionality subscale was 0.735 in the Netherlands, 0.710 in Finland and 0.843 in the UK. For activity, these values were 0.648, 0.697 and 0.832, and for shyness 0.738, 0.784 and 0.760, respectively.

#### Formal child-care characteristics

Three different formal child-care characteristics were examined. First, respondents were asked about the number of hours the target child spent in formal care in the month preceding the survey. Second, respondents were asked in separate questions how many times the target child was in formal care overnight, during early mornings (5–7 am) and during evenings (6–10 pm) during the last month. Because of low variation, these separate questions were combined into one dummy variable indicating whether respondents used formal child care during nonstandard hours (1 = *yes*, 0 = *no*). Third, respondents were asked about the number of different formal care providers they used in the week prior to the survey.

#### Control variables

Several child and family background factors were included in our study to take into account that use of formal child care varies between different groups of parents (Abner et al. 2013) and to minimize the confounding effects of family and child characteristics (Jaffee et al. 2011). This is why, in addition to the respondent’s gender, we control for the family’s financial situation as children of parents with more income are more likely to be enrolled in high-quality child care (Akgündüz and Plantenga 2014). Given known gender differences in problem behavior (Doey et al. 2014; Klein et al. 2013), we also control for the target child’s gender as well as their age (in years) and whether they live with both biological parents, as these factors have been related to behavior problems (Van Zeijl et al. 2006; Waldfogel et al. 2010). Lastly, we include whether the respondent indicated having problems with arranging child care, as this may affect family functioning (Usdansky and Wolf 2008) and therefore child well-being.

### Data Analyses

Descriptive statistics were calculated for the dependent and independent variables, separately per country, after which mean difference tests were executed to examine country differences. Next, children’s socioemotional well-being was predicted using two separate sets of multivariate hierarchical OLS regression models, one for the SDQ subscales for children aged three and over, and one for the EAS subscales for younger children. For both age groups, our first model includes only formal care characteristics. Background factors were added in the second model, and country dummies in the third model. In subsequent models, interactions between formal care characteristics and country were entered separately per formal care characteristic, including the amount of time in care, the scheduling of the hours spent in care and the number of care arrangements. All data analysis was completed using SPSS, version 23 (IBM Corp 2015).

## Results

Descriptive findings show that Finnish parents reported higher levels of externalizing behavior for their target child compared to Dutch parents (Table 3). Concerning prosocial behavior, higher levels are reported in the Netherlands and in the UK compared to Finland. The EAS measure also reveals country differences, with Finnish parents reporting higher levels of emotionality compared to British and Dutch parents. British parents, on the other hand, report higher shyness levels than their Dutch and Finnish counterparts. The dependent variables thus show considerable country differences.

**Table 3:** Descriptive statistics for child well-being and formal child care variables (*N* *=* 990)

	NL (*n* = 318)		FI (*n* = 359)		UK (*n* = 313)		Mean difference test
	*M* (SD)	Range	*M* (SD)	Range	*M* (SD)	Range	
SDQ—Internalizing problem behavior	2.58 (2.57)	0–14	2.72 (2.38)	0–12	2.88 (2.64)	0–14	*ns*
SDQ—Externalizing problem behavior	4.22 (3.44)	0–17	5.61 (3.47)	0–20	4.98 (3.59)	0–17	FI > NL^***^
SDQ—Prosocial behavior	8.06 (1.78)	3–10	7.48 (1.85)	2–10	8.13 (1.73)	4–10	NL & UK > FI^***^
EAS—Emotionality	10.75 (3.04)	5–25	13.11 (3.50)	7–24	11.53 (3.81)	5–25	FI > NL & UK^***^
EAS—Activity	9.24 (2.36)	5–15	9.54 (2.82)	5–18	9.26 (3.36)	5–22	*ns*
EAS—Shyness	10.63 (3.11)	5–19	10.34 (3.06)	5–18	11.97 (3.40)	5–23	UK > NL & FI^**^
Monthly hours in formal care	58.79 (41.15)	3–250	104.41 (51.49)	4–246	74.29 (54.71)	2–294	FI > UK > NL^***^
Formal care during nonstandard hours (1 = *yes*)	0.12	0–1	0.50	0–1	0.05	0–1	FI > NL > UK^***^
# formal care providers	1.13 (0.36)	1–3	1.31 (0.54)	1–4	1.31 (0.53)	1–3	FI & UK > NL^***^

SD is not reported for dichotomous variables

*SDQ* strengths and difficulties questionnaire, *EAS* emotionality activity shyness

**p < 0.01. ***p < 0.001

Even though our sample was restricted to parents of children in formal child care, the descriptives show considerable country differences in the extent that formal care is being used. Finnish parents utilize formal care for the largest number of hours per month, followed by British parents, with Dutch parents reporting the least use of formal care. Moreover, Finnish parents use child care during nonstandard hours more frequently than Dutch parents do, whereas this is relatively uncommon in the British sample. Closer examination of the data reveals that in Finland and in the Netherlands this includes a mixture of overnight, early morning and evening care, whereas in the UK this only consists of early morning and evening care. Furthermore, parents in the UK and Finland use a higher number of different formal care providers than Dutch parents.

In Table 4, the results of the multivariate analyses are presented for children aged three and older, relating different characteristics of formal child care to internalizing, externalizing, and prosocial behavior. Model 1, which includes the formal care characteristics but not the background factors, shows that across all three countries, spending time in formal care during nonstandard hours is associated with more internalizing behavior. Furthermore, spending more time in formal care on a monthly basis is significantly associated with more externalizing behavior (Model 1). When background factors are included in the model, only the association between longer monthly hours and externalizing behavior remains significant (Model 2). This association, however, becomes not significant when the country dummies are included in Model 3, in which Finland constitutes the reference category. Inclusion of these country dummies furthermore reveals that Finnish children only differ significantly from Dutch and British children on prosocial behavior. In order to assess whether there is a significant difference between responses from Dutch and British parents, we changed the reference category to the UK (results not reported in Table 4), which revealed no differences between the Netherlands and the UK. The results for the background factors are in line with prior literature, indicating that children from better financial backgrounds display less internalizing and externalizing behavior, and more prosocial behavior. Moreover, girls display both less externalizing and more prosocial behavior, whereas older children show more internalizing and prosocial behavior.

**Table 4:** Summary of multivariate OLS regression analyses for variables that predict internalizing, externalizing, and prosocial behavior in children aged 3 to 12 (*N* = 684)

	*Model 1*			*Model 2*			*Model 3*		
	Internalizing	Externalizing	Prosocial	Internalizing	Externalizing	Prosocial	Internalizing	Externalizing	Prosocial
	*B (SE)*	*B (SE)*	*B (SE)*	*B (SE)*	*B (SE)*	*B (SE)*	*B (SE)*	*B (SE)*	*B (SE)*
Monthly hours in formal care	−0.00 (0.00)	0.01 (0.00)^*^	−0.00 (0.00)	−0.00 (0.00)	0.01 (0.00)^*^	−0.00 (0.00)	0.00 (0.00)	0.01 (0.00)	0.00 (0.00)
Formal care during nonstandard hours	0.57 (0.26)^*^	0.51 (0.37)	−0.21 (0.19)	0.35 (0.26)	0.28 (0.36)	−0.11 (0.19)	0.46 (0.28)	0.09 (0.40)	0.19 (0.20)
# formal care providers	−0.17 (0.20)	0.22 (0.28)	0.20 (0.14)	−0.29 (0.20)	0.11 (0.28)	0.17 (0.14)	−0.30 (0.20)	0.06 (0.28)	0.19 (0.14)
Family’s financial situation				−0.27 (0.05)^***^	−0.28 (0.07)^***^	0.12 (0.04)^**^	−0.26 (0.05)^***^	−0.27 (0.07)^***^	0.12 (0.04)^**^
Gender respondent (1 = female)				0.37 (0.28)	0.18 (0.39)	0.13 (0.20)	0.37 (0.28)	0.19 (0.39)	0.12 (0.20)
Gender child (1 = girl)				−0.24 (0.21)	−0.79 (0.29)^**^	0.47 (0.15)^**^	−0.22 (0.21)	−0.81 (0.29)^**^	0.51 (0.15)^**^
Age child				0.15 (0.06)^*^	−0.02 (0.08)	0.11 (0.04)^*^	0.15 (0.06)^*^	−0.03 (0.08)	0.11 (0.04)^*^
Child lives with both biological parents				−0.37 (0.32)	0.16 (0.45)	0.13 (0.23)	−0.39 (0.32)	0.19 (0.45)	0.07 (0.23)
Problems with arranging child care				−0.25 (0.23)	0.54 (0.32)	−0.02 (0.17)	−0.33 (0.24)	0.44 (0.34)	−0.04 (0.18)
Country^a^—NL							0.03 (0.30)	−0.71 (0.42)	0.65 (0.22)^**^
Country^a^—UK							0.33 (0.29)	−0.34 (0.41)	0.72 (0.21)^**^
Constant	3.07 (0.32)^***^	4.07 (0.45)^***^	7.79 (0.23)^***^	4.13 (0.65)^***^	5.85 (0.91)^***^	6.07 (0.47)^***^	4.03 (0.69)^***^	6.40 (0.97)^***^	5.47 (0.50)^***^
R²	0.01	0.02	0.01	0.07	0.06	0.05	0.07	0.06	0.07

*p < 0.05. **p < 0.01. ***p < 0.001

^a^Reference = Finland

Table 5 presents the results of the models after adding interaction terms between the formal care characteristics and country dummies. Results revealed no significant differences between Finland and the Netherlands or the UK regarding internalizing problem behavior (with Finland as the reference category). Changing the reference category to the UK, however, revealed that the effect of spending longer monthly hours in formal care differs between the UK and the Netherlands. Spending more time in formal care is associated with more internalizing behavior in the Netherlands compared to the UK (*B* *=* 0.01, *p* = 0.044), which is partly in line with our first hypothesis. No other significant interactions were found, also not for externalizing or prosocial behavior.

**Table 5:** Summary of multivariate OLS regression analyses for variables that predict internalizing, externalizing, and prosocial behavior, including interaction terms between formal child care and country (*N* = 684)

	Internalizing	Externalizing	Prosocial
	*B (SE)*	*B (SE)*	*B (SE)*
*Model 4*			
Monthly hours in formal care	0.00 (0.00)	0.01 (0.00)	0.00 (0.00)
Country^a^—NL	0.07 (0.31)	−0.73 (0.44)	0.67 (0.23)^**^
Country^a^—UK	0.26 (0.29)	−0.25 (0.41)	0.71 (0.21)^**^
Monthly hours * NL	0.01 (0.01)^b^	−0.01 (0.01)	0.00 (0.00)
Monthly hours * UK	−0.00 (0.01)	0.00 (0.01)	−0.00 (0.00)
Constant	4.01 (0.71)^***^	6.38 (1.00)^***^	5.45 (0.51)^***^
*Model 5*			
Formal care during nonstandard hours	0.60 (0.34)	0.61 (0.47)	0.19 (0.14)
Country^a^—NL	0.11 (0.33)	−0.44 (0.46)	0.63 (0.25)^**^
Country^a^—UK	0.42 (0.31)	−0.04 (0.43)	0.71 (0.22)^**^
Care during nonstandard hours * NL	−0.29 (0.74)	−1.45 (1.04)	0.12 (0.53)
Care during nonstandard hours * UK	−0.79 (0.91)	−2.15 (1.28)	−0.02 (0.65)
Constant	3.94 (0.70)^***^	6.07 (0.99)^***^	5.49 (0.51)^***^
*Model 6*			
# of formal care providers	0.00 (0.29)	0.25 (0.41)	0.20 (0.21)
Country^a^—NL	0.79 (0.75)	−0.23 (1.06)	0.77 (0.54)
Country^a^—UK	1.10 (0.67)	0.14 (0.96)	0.70 (0.49)
# of formal care providers * NL	−0.60 (0.56)	−0.39 (0.79)	−0.10 (0.40)
# of formal care providers * UK	−0.56 (0.43)	−0.34 (0.61)	0.01 (0.31)
Constant	3.64 (0.74)^***^	6.16 (1.05)^***^	5.46 (0.53)^***^

The regressions presented in this table include the same set of independent and control variables that were included in the regressions in Table 3, Model 3

**p < 0.01. ***p < 0.001

^a^Reference = Finland

^b^*p*  < 0.05 (compared to UK)

Table 6 presents the results of the multivariate analyses for children aged up to three years old, concerning the association between formal child care and levels of emotionality, activity, and shyness. When only formal care characteristics are included (Model 1), results show that increases in the number of care providers are associated with higher emotionality. None of the formal care characteristics were significantly associated with activity or shyness. After adding background factors in Model 2, the association between the number of care providers and emotionality remains significant. However, this is no longer the case once country dummies are included in the analyses in Model 3. This model does reveal, however, that Dutch parents report lower levels of their child’s emotionality than Finnish parents. Furthermore, British children show higher levels of shyness compared to Finnish children. No significant country differences were found for children’s activity levels. Changing the reference category to the UK revealed no additional country differences. Results of the background factors are in line with prior literature cited above, indicating that girls show higher levels of shyness and that younger children show more emotionality and shyness.

**Table 6:** Summary of multivariate OLS regression analyses for variables that predict emotionality, activity, and shyness in children aged 0 to 2 (*N* = 306)

	*Model 1*			*Model 2*			*Model 3*		
	Emotionality	Activity	Shyness	Emotionality	Activity	Shyness	Emotionality	Activity	Shyness
	*B (SE)*	*B (SE)*	*B (SE)*	*B (SE)*	*B (SE)*	*B (SE)*	*B (SE)*	*B (SE)*	*B (SE)*
Monthly hours in formal care	0.01 (0.01)	0.01 (0.00)	0.00 (0.00)	0.00 (0.01)	0.01 (0.00)	0.00 (0.00)	0.00 (0.01)	0.01 (0.00)	0.00 (0.01)
Formal care during nonstandard hours	0.81 (0.56)	0.22 (0.44)	−0.66 (0.52)	0.66 (0.57)	0.44 (0.45)	−0.50 (0.53)	0.05 (0.64)	0.39 (0.51)	0.27 (0.58)
# formal care providers	1.63 (0.63)^*^	−0.08 (0.50)	0.28 (0.59)	1.26 (0.63)^*^	−0.07 (0.51)	0.15 (0.59)	1.10 (0.63)	−0.06 (0.51)	0.24 (0.58)
Family’s financial situation				−0.12 (0.11)	0.13 (0.09)	−0.01 (0.10)	−0.12 (0.12)	0.12 (0.09)	0.04 (0.11)
Gender respondent (1 = female)				−0.34 (0.72)	−0.16 (0.58)	−0.61 (0.67)	−0.38 (0.72)	−0.15 (0.58)	−0.58 (0.66)
Gender child (1 = girl)				0.24 (0.44)	0.39 (0.35)	0.97 (0.41)^*^	0.25 (0.44)	0.37 (0.35)	1.04 (0.40)^*^
Age child				0.89 (0.33)^**^	0.26 (0.26)	0.55 (0.30)	0.72 (0.34)^*^	0.29 (0.27)	0.62 (0.31)^*^
Child lives with both biological parents				−1.17 (0.83)	0.60 (0.66)	0.29 (0.77)	−1.10 (0.84)	0.64 (0.67)	0.08 (0.77)
Problems with arranging child care				0.85 (0.55)	0.80 (0.44)	1.00 (0.51)	0.81 (0.58)	0.90 (0.46)	0.68 (0.53)
Country^a^—NL							−1.56 (0.68)^*^	0.14 (0.55)	1.00 (0.63)
Country^a^—UK							−1.42 (0.72)	−0.17 (0.58)	1.97 (0.67)^**^
Constant	9.00 (0.83)^***^	8.94 (0.65)^***^	10.45 (0.76)^***^	10.20 (1.70)^***^	6.87 (1.35)^***^	9.38 (1.57)^***^	12.05 (1.86)^***^	6.79 (1.50)^***^	7.88 (1.71)^***^
R²	0.03	0.01	0.01	0.06	0.04	0.06	0.07	0.04	0.09

*p < 0.05. **p < 0.01. ***p < 0.001

^a^Reference = Finland

Next, we included interaction terms between formal care characteristics and country dummies. Model 5 in Table 7 reveals that children who are in formal care during nonstandard hours in the UK show lower emotionality levels than their Finnish counterparts. This is in contrast with our third hypothesis. No significant interaction terms were found for children’s activity levels. With respect to shyness, no differences were found between Finland and the Netherlands or the UK. Changing the reference category to the UK revealed that Dutch children who receive care from more care providers show lower shyness levels than British children (*B* = −3.57, *p* = 0.009), which partly contradicts our third hypothesis.

**Table 7:** Summary of multivariate OLS regression analyses for variables that predict emotionality, activity, and shyness, with interaction terms between formal child care and country (*N* = 306)

	Emotionality	Activity	Shyness
	*B (SE)*	*B (SE)*	*B (SE)*
*Model 4*			
Monthly hours in formal care	0.01 (0.01)	0.01 (0.01)	0.01 (0.01)
Country^a^—NL	−1.55 (0.79)	0.38 (0.64)	1.08 (0.73)
Country^a^—UK	−1.29 (0.77)	−0.09 (0.62)	2.05 (0.71)^**^
Monthly hours * NL	0.01 (0.01)	−0.01 (0.01)	−0.00 (0.01)
Monthly hours * UK	−0.01 (0.01)	−0.00 (0.01)	−0.00 (0.01)
Constant	11.83 (2.08)^***^	6.37 (1.67)^***^	7.63 (1.92)^***^
*Model 5*			
Formal care during nonstandard hours	1.45 (1.02)	−0.74 (0.82)	0.61 (0.94)
Country^a^—NL	−0.67 (0.86)	−0.58 (0.70)	1.26 (0.80)
Country^a^—UK	−0.50 (0.86)	−0.87 (0.69)	2.10 (0.79)^**^
Care during nonstandard hours * NL	−1.67 (1.32)	1.56 (1.07)	−0.89 (1.22)
Care during nonstandard hours * UK	−5.66 (2.30)^*^	3.27 (1.86)	1.50 (2.13)
Constant	10.76 (1.94)^***^	7.73 (1.57)^***^	7.79 (1.80)^***^
*Model 6*			
# of formal care providers	1.41 (1.20)	−0.33 (0.97)	0.03 (1.09)
Country^a^—NL	−0.53 (1.97)	0.48 (1.58)	2.42 (1.79)
Country^a^—UK	−1.54 (2.06)	−1.59 (1.66)	−0.51 (1.87)
# of formal care providers * NL	−0.91 (1.58)	−0.35 (1.27)	−1.35 (1.44)^b^
# of formal care providers * UK	0.15 (1.62)	1.25 (1.30)	2.22 (1.47)
Constant	11.67 (2.25)^***^	7.13 (1.80)^***^	8.14 (2.04)^***^

The regressions presented in this table include the same set of independent and control variables that were included in the regressions in Table 5, Model 3

*p < 0.05. **p < 0.01. ***p < 0.001

^a^Reference = Finland

^b^*p* < 0.01 (compared to UK)

## Discussion

As changes in maternal employment patterns have prompted many parents to reconsider their child-care arrangements, the use of formal child care has expanded rapidly in Western countries. According to Bronfenbrenner’s ecological systems theory (1979), individuals are affected by interactions in their microsystems (e.g., peers, family, educational settings). This implies that nonparental relationships, i.e., in formal child-care settings, matter for children’s socioemotional development. We extended prior research on the association between formal child care and children’s socioemotional well-being by comparing countries that differ regarding family policies. More specifically, we examined whether children’s socioemotional well-being was differently affected by the number of monthly hours spent in formal care, the use of formal care during nonstandard hours and the number of different care providers depending on the country context. We hypothesized that formal child care is associated with better child well-being outcomes in countries in which policies are targeted towards high-quality care. Among the countries included in our study, Finland ranks the highest with regards to the overall quality of child care and we therefore expected to see better socioemotional child outcomes in Finland, compared to the Netherlands and the UK. Findings indicate that there are indeed differences in the socioemotional well-being of Dutch, Finnish and British children, and that these differences were partly related to country differences in formal care characteristics.

Before discussing the findings in more detail, some caution is warranted regarding the strength of our findings. As the analyses involved testing a large number of associations, including the interactions, the likelihood for a Type I error increased (Šimundić 2013). In other words, the probability of finding an interaction that is in reality spurious may have been elevated. This should be kept in mind when interpreting the three main findings that are discussed below.

First, in line with our expectations, longer monthly hours in formal care were less beneficial for Dutch children than for British children, given the stronger association with internalizing behavior in the Netherlands than in the UK. The lower-quality care in the Netherlands, as indicated by the Starting Well Index (Watson 2012), may explain some of these differences, but social norms concerning formal child care may also matter. Although formal child care is becoming increasingly accepted in the UK (Fagan and Norman 2012), it is still viewed with some suspicion in the Netherlands (Merens and Van den Brakel 2014). Perhaps the normative context in which our respondents lived influenced how they evaluated their children’s behavior, with Dutch parents being more worried about the effects of formal child care and thus more likely to report internalizing behavior (cf. Duncan and Edwards 2003, on the ‘moral rationalities’ that parents use to make decisions about child care and to evaluate these).

Second, Finnish children who spent time in formal care during nonstandard hours showed higher levels of emotionality compared to British children, which was contrary to our expectations. However, further inspection of the data revealed that whereas British children mainly spent early mornings or evenings in formal care during nonstandard hours, Finnish children spent more nights in formal care. This finding is in line with previous research on care during nonstandard hours (Anme and Segal 2003; Boyd-Swan 2015) and may point to the disruptive consequences of overnight care. However, it could also indicate that parents who leave their children in overnight care may be more inclined to make note of any problematic behavior because they are primed to do so in the context of heated public debates held in Finland concerning the effects of overnight care on children. The potential negative consequences of night shifts have also been discussed widely in societal debate (e.g., Williams 2016), which may further invoke negative feelings among parents working night shifts towards their child-care arrangements. Therefore, these parents may more readily notice and attribute a pattern to their child’s negative behavior than parents who use child care during regular hours.

Third, having more care providers was more strongly related to shyness levels among British children than among Dutch children. This finding was in contrast to our expectations, as we hypothesized that increases in the number of caregivers would be most harmful for Dutch children. Yet, our study revealed that British children are confronted with more caregivers than Dutch children. Scholars have argued that experiencing multiple care arrangements makes it hard for children to build relationships with their caregivers and peers (Claessens and Chen 2013; Morrissey 2009), which hampers children’s social skills. This may explain why British children, who, on average, experience higher instability in formal care, show higher shyness levels than Dutch children. In contrast, higher shyness among British children may also be a country characteristic, as British people are often portrayed as being very reserved (Harley 2003).

### Limitations and Future Research

Despite the valuable contribution of this study of applying a comparative approach to examine the association between formal care characteristics and child well-being, some limitations need to be mentioned. First, the respondents of our web survey were not randomly selected; therefore, our sample may not be representative of the populations in the countries under study or of the parents who typically use formal child care in these countries. Parents self-selected into the study and it is therefore possible that our sample contains respondents that were particularly motivated to participate. Also, Finnish nonstandard workers were overrepresented, which may as least partly explain the differences we found between countries. However, as comparative studies on this subject are scarce, we believe we have taken an important first step in providing insight into the extent to which the characteristics of formal child care may affect children differently depending on the country context. We encourage researchers to continue this line of research, especially when a randomly selected cross-country dataset should become available. Second, our data were based on one source, namely parents’ self-reports of their children’s behavior, which brings along the risk of socially desirable answers, especially on a sensitive topic like children’s socioemotional well-being. It would have been desirable to supplement these reports with evaluations from child-care workers to support the validity of our findings. However, both the SDQ and the EAS have shown good inter-rater agreement when comparing parents and teachers (Gasman et al. 2002; Stone et al. 2010), which points to the validity of parents’ reports. Moreover, some of our multi-item measures displayed Cronbach’s alpha scores which showed questionable rather than acceptable internal consistency. Lastly, we assume that country effects are related to family policy differences. However, because we did not measure these policies directly we cannot draw definite conclusions on this topic. Examining this topic with a larger set of countries, which would allow for inclusion of country-level factors, is therefore desirable (Yu 2015). Moreover, our data were collected at one point in time, which does not allow us to investigate changes over time following changes in policies. The country dummies that were included in the analysis may therefore capture a number of differences in macrolevel conditions—such as family policies, social norms, employment conditions, the percentage of GPD spent on early education, etc. The nature of our data does not allow us to speculate which of the macro variables is most important in explaining the country differences that we observe in our results.

Future research should aim to further investigate associations between child-care quality and child outcomes across a number of countries and time periods in order to better understand the role of country-specific conditions, including family policies, macroeconomic conditions and social norms. Nationally representative data collected over a number of years in multiple countries would allow researchers to better assess the influence of various country-level variables. The child-care sector is dynamic, which is visible in recent changes in this sector in the countries under study (British Department for Education 2013; Finlex 2015; Social and Economic Council of the Netherlands 2016). One aspect of formal care that gains limited emphasis within these policy discussions seems to be care during nonstandard hours, although this applies to a lesser extent to Finland. Nonetheless, given the findings of prior research, combined with the results of the current study, we believe that more attention should be paid to how care during nonstandard hours may affect children, especially in light of continuing increases in nonstandard work schedules (Bünning and Pollmann-Schult 2016; Presser 2003). Providing high-quality formal care during nonstandard hours will not only benefit children, but also parents themselves, as they will more easily be able to reconcile work and family obligations.

In sum, our study demonstrated that in all three countries, formal child care was associated with children’s socioemotional well-being, but our findings also showed country differences in this association. This demonstrates the benefit of taking a comparative perspective in this line of research and we therefore encourage researchers to continue on this path by extending research into formal child care to include countries that offer less support to parents in reconciling work and family obligations. Even though the Netherlands, Finland and the UK differ in their family policies, these countries can all generally be considered as supportive in allowing parents to combine work and family obligations. Therefore, it would be interesting to examine how the countries under study compare with countries with less supportive family policies (see, for example, Korpi 2000). Special attention should be paid to care during nonstandard hours. Given the increasing prevalence of nonstandard work, the issue of overnight care—how to provide this and deliver high-quality care—is likely to become a pressing issue for parents and policymakers alike.

## References

[CR1] Abner KS, Gordon Ra, Kaestner R, Korenman S (2013). Does child-care quality mediate associations between type of care and development?. Journal of Marriage and Family.

[CR2] Akgündüz YE, Plantenga J, Gambaro L, Stewart K, Waldfogel J (2014). Equal access to high-quality childcare in the Netherlands. An equal start? Providing quality early education and care for disadvantaged children.

[CR3] Alila, K. (2015). *Provision of Quality Early Childcare Services – Finland*. European Commission, Comments Paper. http://ec.europa.eu/social/BlobServlet?docId=14819&langId=en.

[CR4] Anme T, Segal UA (2003). Center-based evening child care: implications for young children’s development. Early Childhood Education Journal.

[CR5] Boogaard M, Bollen I (2014). Gastouders in beeld: Een inventarisatiestudie onder gastouders in Nederland [Portraying childminders: An inventory study among childminders in the Netherlands]..

[CR6] Boyd-Swan, C. H. (2015). *Nonparental child care during nonstandard hours: Who uses it and how does it influence child well-being?* (Doctoral Dissertation, Arizona State University).

[CR7] British Department for Education. (2013). More great childcare: raising quality and giving parents more choice..

[CR8] Broekhuizen ML, Mokrova IL, Burchinal MR, Garrett-Peters P (2016). Classroom quality at pre-kindergarten and kindergarten and children’s social and behavioral skills. Early Childhood Research Quarterly.

[CR9] Bronfenbrenner U (1979). The ecology of human development: experiments by nature and design..

[CR10] Bronfenbrenner U, Ceci SJ (1994). Nature-nuture reconceptualized in developmental perspective: a bioecological model. Psychological Review.

[CR11] Bünning M, Pollmann-Schult M (2016). Parenthood, child care, and nonstandard work schedules in Europe. European Societies.

[CR12] Buss AH, Plomin R (1984). Temperament: early developing personality traits..

[CR13] Campbell F, Conti G, Heckman JJ, Moon SH, Pinto R, Pungello E, Pan Y (2014). Early childhood investments substantially boost adult health. Science.

[CR14] Claessens A, Chen JH (2013). Multiple child care arrangements and child well being: Early care experiences in Australia. Early Childhood Research Quarterly.

[CR15] Crompton R, Lewis S, Lyonette C (2007). Women, men, work and family in Europe.

[CR16] De Hond, K., Remery, C. L. H. S., Tissing, H. A., & Zeeman, S. (2012). Onderzoek naar de kwaliteit van kinderopvang in EU-/EER-landen [Research on the quality of formal child care in EU-/EER countries]. http://www.bureaubartels.nl/site/assets/files/1254/0476_kwaliteit-van-kinderopvang-in-eu-eer-landen.pdf.

[CR17] De Jong, M. (2013, February 20). Mama draait een nachtdienst, kind logeert bij opvang [Mom works the night shift, child stays in formal child care]. *Nieuw Nederlands Peil*. https://www.napnieuws.nl/2013/02/20/mama-draait-een-nachtdienst-kroost-logeert-bij-opvang/.

[CR18] Doey L, Coplan RJ, Kingsbury M (2014). Bashful boys and coy girls: a review of gender differences in childhood shyness. Sex Roles.

[CR19] Duncan S, Edwards R, Kollind K, Peterson A (2003). State welfare regimes, mothers’ agencies and gendered moral rationalities. Thoughts on family, gender, generation and class. A Festschrift to Ulla Björnberg.

[CR20] Dutch Child Care Act. (2016). no. AV/KO/2004/65638.

[CR21] Dutch Government. (2014). Welke vormen van kinderopvang zijn er? [Which types of child care exist?]. https://www.rijksoverheid.nl/onderwerpen/kinderopvang/vraag-en-antwoord/soorten-kinderopvang.

[CR22] EACEA. (2009). Early childhood education and care in Europe: tackling social and cultural inequalities: The Netherlands.

[CR23] Engster D, Stensöta HO (2011). Do family policy regimes matter for children’s well-being?. Social Politics.

[CR24] Fagan C, Norman H (2012). Trends and social divisions in maternal employment patterns following maternity leave in the UK. International Journal of Sociology and Social Policy.

[CR25] Finlex. (2015). Hallituksen esitys eduskunnalle laeiksi varhaiskasvatuslain sekä lasten kotihoidon ja yksityisen hoidon tuesta annetun lain muuttamisesta [The Government’s proposal to amend the laws on early childhood education, home care and the state subsidy for private child care], HE 80/2015 vp. http://www.finlex.fi/fi/esitykset/he/2015/20150080.

[CR26] Finlex. (2016). Basic Education Act 628/1998, Amendment 1136/2003. http://www.finlex.fi/en/laki/kaannokset/1998/en19980628.pdf.

[CR27] Finnish National Agency for Education. (2016). Early childhood education and care. https://www.oph.fi/english/education_system/early_childhood_education.

[CR28] Formby, E., Tang, N., & Yeandle, S. (2004). Supporting work-life balance using non-standard hours childcare. http://www.sociology.leeds.ac.uk/assets/files/Circle/formby-supporting-wlb.pdf.

[CR29] Fukkink, R. G., Gevers Deynoot-Schaub, M. J. J. M., Helmerhorst, K. O. W., Bollen, I., & Riksen-Walraven, J. M. A. (2013). Pedagogische kwaliteit van de kinderopvang voor 0- tot 4-jarigen in Nederlandse kinderdagverblijven in 2012 [Pedagogical quality of formal child care for 0 to 4 year old children in Dutch child-care settings in 2012]. http://www.kinderopvangonderzoek.nl.

[CR30] Gasman I, Purper-Ouakil D, Michel G, Mouren-Siméoni MC, Bouvard M, Perez-Diaz F, Jouvent R (2002). Cross-cultural assessment of childhood temperament: A confirmatory factor analysis of the French Emotionality Activity and Sociability (EAS) questionnaire. European Child and Adolescent Psychiatry.

[CR31] Goodman A, Lamping DL, Ploubidis GB (2010). When to use broader internalising and externalising subscales instead of the hypothesised five subscales on the strengths and difficulties questionnaire (SDQ): Data from British parents, teachers and children. Journal of Abnormal Child Psychology.

[CR32] Goodman R (1997). The strengths and difficulties questionnaire: a research note. Journal of Child Psychology and Psychiatry.

[CR33] Greenberg JP (2010). Assessing policy effects on enrollment in early childhood education and care. Social Service Review.

[CR34] Griggs MS, Gagnon SG, Huelsman TJ, Kidder‐Ashley P, Ballard M (2009). Student–teacher relationships matter: moderating influences between temperament and preschool social competence. Psychology in the Schools.

[CR35] Hamilton M, Redmond G (2010). Conceptualisation of social and emotional wellbeing for children and young people, and policy implications.

[CR36] Harley TA, Strauss S, Orlove BS (2003). Nice weather for the time of year: The British obsession with the weather. Weather, climate, culture.

[CR37] Howes C, Smith PK, Hart CH (2011). Children’s social development within the socialization context of child care and early childhood education. The Wiley-Blackwell handbook of childhood social development.

[CR38] IBM Corp. (2015). IBM SPSS statistics for windows, version 23.0.

[CR39] Jaffee SR, Van Hulle C, Rodgers JL (2011). Effects of nonmaternal care in the first 3years on children’s academic skills and behavioral functioning in childhood and early adolescence: a sibling comparison study. Child Development.

[CR40] Klein AM, Otto Y, Fuchs S, Zenger M, Von Klitzing K (2013). Psychometric properties of the parent-rated SDQ in preschoolers. European Journal of Psychological Assessment.

[CR41] Korpi W (2000). Faces of inequality: gender, class, and patterns of inequalities in different types of welfare states. Social Politics.

[CR42] Kröger T (2010). Lone mothers and the puzzles of daily life: Do care regimes really matter?. International Journal of Social Welfare.

[CR43] Lamb ME, Ahnert L, Damon W, Lerner RM, Renninger KA, Sigel IE (2006). Nonparental child care: context, concepts, correlates, and consequences. Handbook of child psychology (Vol. 4) Child psychology in practice.

[CR44] Lloyd E, Penn H (2010). Why do childcare markets fail?: Comparing England and the Netherlands. Public Policy Research.

[CR45] Loeb S, Bridges M, Bassok D, Fuller B, Rumberger RW (2007). How much is too much? The influence of preschool centers on children’s social and cognitive development. Economics of Education Review.

[CR46] Magnuson KA, Ruhm C, Waldfogel J (2007). Does prekindergarten improve school preparation and performance?. Economics of Education Review.

[CR47] Mahon R (2002). Child care: Toward what kind of “social Europe”?. Social Politics.

[CR48] Mamolo M, Coppola L, Di Cesare M (2011). Formal childcare use and household socio-economic profile in France Italy, Spain and UK. Population Review.

[CR49] McCartney K, Burchinal M, Clarke-Stewart A, Bub KL, Owen MT, Belsky J, the NICHD Early Child Care Research Network. (2010). Testing a series of causal propositions relating time in child care to children’s externalizing behavior. Developmental Psychology.

[CR50] Melhuish, E., Ereky-Stevens, K., Petrogiannis, K., Ariescu, A., Penderi, E., Rentzou, K.,… & Leseman, P. (2015). A review of research on the effects of Early Childhood Education and Care (ECEC) upon child development. http://ecec-care.org/resources/publications/.

[CR51] Merens A, Van den Brakel M (2014). Emancipatiemonitor 2014 [Emancipation monitor 2014].

[CR52] Moilanen S, May V, Räikkönen E, Sevón E, Laakso ML (2016). Mothers’ non-standard working and childcare-related challenges: A comparison between lone and coupled mothers. International Journal of Sociology and Social Policy.

[CR53] Morgan KJ, Zippel K (2003). Paid to care: the origins and effects of care leave policies in Western Europe. Social Politics.

[CR54] Morrissey TW (2009). Multiple child care arrangements and young children’s behavioral outcomes. Child Development.

[CR55] NCT. (2016). Childcare options. https://www.nct.org.uk/parenting/childcare-options.

[CR56] Nores M, Barnett WS (2010). Benefits of early childhood interventions across the world: (Under) Investing in the very young. Economics of Education Review.

[CR57] Nutbrown. (2012). *Foundations for quality: the independent review of early education and childcare qualifications*. https://www.gov.uk/government/uploads/system/uploads/attachment_data/file/175463/Nutbrown-Review.pdf.

[CR58] OECD. (2016). Education at a glance 2016: OECD Indicators..

[CR59] OECD Family Database. (2014). Enrolment in childcare and pre-school. http://www.oecd.org/social/family/database.htm.

[CR60] OECD Family Database. (2015). Childcare support. http://www.oecd.org/social/family/database.htm.

[CR61] Pakarinen E, Lerkkanen MK, Poikkeus AM, Kiuru N, Siekkinen M, Rasku Puttonen H, Nurmi JE (2010). A validation of the classroom assessment scoring system in Finnish kindergartens. Early Education & Development.

[CR62] Phillips D, Adams G (2001). Child care and our youngest children. The Future of Children.

[CR63] Presser HB (2003). Working in a 24/7 economy.

[CR64] Rigby E, Ryan RM, Brooks-Gunn J (2007). Child care quality in different state policy contexts. Journal of Policy Analysis and Management.

[CR65] Säkkinen S (2014). Lasten päivähoito 2013 – kuntakyselyn osaraportti [Children’s daycare 2013 – statistical report of municipal survey].

[CR66] Šimundić AM (2013). Bias in research. Biochemia Medica.

[CR67] Social and Economic Council of the Netherlands. (2016). Gelijk goed van start [Immediately a good start]..

[CR68] Stone LL, Otten R, Engels RCME, Vermulst AA, Janssens JMAM (2010). Psychometric properties of the parent and teacher versions of the strengths and difficulties questionnaire for 4- to 12-year-olds: a review. Clinical Child and Family Psychology Review.

[CR69] Usdansky ML, Wolf DA (2008). When child care breaks down: mothers’ experiences with child care problems and resulting missed work. Journal of Family Issues.

[CR70] Vandell DL, Belsky J, Burchinal M, Steinberg L, Vandergrift N, the NICHD Early Child Care Research Network. (2010). Do effects of early child care extend to age 15 years: results from the NICHD study of early child care and youth development. Child Development.

[CR71] Van Hooren F, Becker U (2012). One welfare state, two care regimes: understanding developments in child and elderly care policies in the Netherlands. Social Policy and Administration.

[CR72] Van Zeijl J, Mesman J, Stolk MN, Alink LRA, van IJzendoorn MH, Bakermans-Kranenburg MJ, Koot HM (2006). Terrible ones? Assessment of externalizing behaviors in infancy with the Child Behavior Checklist. Journal of Child Psychology and Psychiatry and Allied Disciplines.

[CR73] Verhoef M, Tammelin M, May V, Rönkä A, Roeters A (2016). Childcare and parental work schedules: a comparison of childcare arrangements among Finnish, British and Dutch dual-earner families. Community, Work & Family.

[CR74] Votruba-Drzal E, Coley RL, Koury AS, Miller P (2013). Center-based child care and cognitive skills development: Importance of timing and household resources. Journal of Educational Psychology.

[CR75] Waldfogel J, Craigie TA, Brooks-Gunn J (2010). Fragile families and child wellbeing. The Future of Children.

[CR76] Watson, J. (2012). *Starting well. Benchmarking early education across the world*. *A report from the Economist Intelligence Unit*. A report from the Economist Intelligence Unit. Singapore: Lien Foundation..

[CR77] Weiland C, Yoshikawa H (2013). Impacts of a prekindergarten program on children’s mathematics, language, literacy, executive function, and emotional skills. Child Development.

[CR78] Williams, S. (2016, January 11). Mothers who leave their children at nursery ALL NIGHT: it’s almost beyond belief. But as women face pressure to work ever longer hours, some are taking a shocking step. *The Daily Mail*. http://www.dailymail.co.uk/femail/article-3392999/Mothers-leave-children-nursery-NIGHT-s-belief-women-10.1007/s10826-018-1185-2face-pressure-work-longer-hours-taking-shocking-step.html.

[CR79] Wincott D (2006). Paradoxes of new labour social policy: toward universal child care in Europe’s “most liberal” welfare regime?. Social Politics.

[CR80] Yu WH (2015). Placing families in context: challenges for cross‐national family research. Journal of Marriage and Family.

[CR81] Zinsser C (2001). Child care within the family. The Future of Children.

